# Importance of Multifaceted Approaches in Infection Control: A Practical Experience from an Outbreak Investigation

**DOI:** 10.1371/journal.pone.0157981

**Published:** 2016-06-20

**Authors:** Nina Katharina Stock, Petr Petráš, Oto Melter, Gabriela Kapounová, Petra Vopalková, Jan Kubele, Václav Vaniš, Jan Tkadlec, Eva Bukáčková, Ivana Machová, Vlastimil Jindrák

**Affiliations:** 1 National Institute of Public Health (NIPH), Prague, Czech Republic; 2 European Program for Public Health Microbiology (EUPHEM), ECDC, Stockholm, Sweden; 3 Department of Medical Microbiology, 2nd Faculty of Medicine and University Hospital Motol, Prague, Czech Republic; 4 Department of Clinical Microbiology and Antibiotic Centre, Na Homolce Hospital, Prague, Czech Republic; Ella Foundation, INDIA

## Abstract

**Background:**

This study presents the results of a multidisciplinary, nosocomial MRSA outbreak investigation in an 8-bed medical intensive care unit (ICU). The identification of seven MRSA positive patients in the beginning of 2014 led to the closure of the ward for several weeks. A multidisciplinary, retrospective investigation was initiated in order to identify the reason and the source for the outbreak, describe MRSA transmission in the department and identify limitations in infection control.

**Methods:**

The investigation comprised an epidemiological description of MRSA cases from 2012 to 2014 and a characterization of MRSA isolates, including phage-, spa- and PFGE-typing. Additionally, MRSA screening was performed from the hospital staff and the environment. To identify the reason for the outbreak, work-related, psychological and behavioral factors were investigated by impartial audits and staff interviews.

**Results:**

Thirty-one MRSA cases were registered during the study period, and 36 isolates were investigated. Molecular typing determined the outbreak strain (phage type 54/812, PFGE type A4, spa type t003) and identified the probable index case. Nasal carriage in one employee and a high environmental contamination with the outbreak strain was documented. Important gaps in nursing procedures and general management were identified. Elevated stress levels and communication problems preceded the outbreak. Compliance with hand hygiene and isolation procedures was evaluated as appropriate.

**Conclusion:**

This study demonstrates the complexity of controlling hospital-associated infections. The combined use of different typing methods is beneficial for outbreak investigations. Psychological, behavioral and other work-related factors have an important impact on the spread of nosocomial pathogens. These factors should be addressed and integrated in routine infection control practice.

## Introduction

### General background

Methicillin resistant *Staphylococcus aureus* (MRSA) is an important cause of healthcare-associated infections (HAI) worldwide and has a substantial influence on the course of disease, mortality and healthcare costs [1]. Transmission occurs mainly via direct persons-to-person contact or contact with contaminated objects. Cross-transmission by healthcare workers (HCW) can be prevented by consequent adherence to the recommended standard precautions such as hand hygiene practices [2–4]. The occurrence of MRSA is frequently not assessed in a standardised way. The stated prevalence is highly dependent on surveillance, prevention and control activities in place and varies considerably between healthcare institutions. Underestimation is likely and may lead to an increased risk for healthcare-acquired MRSA infections (HA-MRSA) [1,5]. In the Czech Republic (CZ), the average MRSA proportion of invasive *S*. *aureus* isolates has been stable at 13%–15% since 2005, although varying between 0% and 50% among 67 reporting hospitals in 2013 [1]. According to the national guidelines for the prevention and control of MRSA, Czech hospitals follow a risk-based screening strategy (www.cls.cz/dokumenty/dp_mrsa.doc).

Nosocomial MRSA outbreaks should always be taken seriously and an investigation should be performed to stop the outbreak and to identify the source, reasons, specific risk factors and weaknesses in standard infection control processes [6,7]. The minimum investigation includes the outbreak confirmation, a general description of cases and the implementation of immediate control measures in order to stop further transmission. Advanced investigations may include analytical epidemiological studies and microbiological typing techniques, as well as studies from all disciplines which are beneficial for the identification of weaknesses in infection control practices. Communication of the results to medical staff is crucial for education purposes. The use of molecular typing is valuable for outbreak confirmation and for the identification of the source and relevant transmission routes. However, no typing method fulfils universal needs and methodological differences as well as discriminatory power have to be considered [8,9]. Frequently used typing methods for *S*. *aureus* include multilocus sequence typing (MLST), SCC*mec* typing, pulsed-field gel electrophoresis (PFGE) typing, spa typing and formerly phage typing [10,11]. PFGE typing is highly discriminative and frequently used in epidemiological studies of nosocomial infections worldwide.

### Description of the MRSA outbreak situation and immediate control measures

The overall proportion of HA-MRSA from all registered MRSA cases within the hospital described in this study was 21–23% since 2011; 30–50% of all HA-MRSA cases were registered in the affected department.

At the end of January 2014 an increase in MRSA cases was noticed at the intensive care unit (ICU) of the affected department. The first case was notified on 20.01.2014 through routine surveillance testing of tracheal aspirate specimens. A second case was identified on 30.01.2014 through the investigation of a wound swab. MRSA screening of close contacts revealed three more cases among the ICU patients. Consequently, an enhanced MRSA screening was performed on 03.02.2014 with swabs obtained from all ICU patients and their contacts, the environment (14 swabs) and the anterior nares of ICU staff. This screening resulted in overall seven cases among the eight ICU patients, three nasal carriers among the ICU staff (whereof two were previously known MRSA carriers) and four positive environmental samples (portable ultrasound device, sanitation chair, plastic tissue retainer and shampoo flask). MRSA cases were cohort isolated at the ICU and the ward was closed for new admissions on 07.02.14. An additional intensification of hygiene practices and environmental cleaning stopped further transmission. The last case was identified on 08.02.14 at the standard ward.

In order to identify the source and factors that might have caused the outbreak in 2014, a comprehensive retrospective outbreak investigation was initiated. Further aims were to describe MRSA transmission in the affected department and to evaluate the quality of nursing, infection control measures and general work conditions. The investigation considered the time period from 01.01.2012 to 18.02.2014 and pursued three key aspects: i) a descriptive epidemiological analysis of the MRSA cases, ii) a microbiological characterisation of the MRSA isolates and iii) an investigation of psychological and work-related factors.

## Methods

### Description of the outbreak setting, screening and isolation procedures

The described outbreak took place at a medical ICU in a Czech tertiary care hospital with an established infection control program. The affected department comprises of one standard ward (SW) with 21 beds and one ICU with eight beds. The department has 24 doctors and 49 nurses employed permanently.

The ICU is arranged in one 4-bed room with a shared bathroom and two 2-bed rooms sharing another bathroom, all directly accessible from the central nurse station. The core ICU staff comprises of three doctors, 18 nurses and four assistants. The patient population contained a high proportion of chronically ill and polymorbid patients with an average length of stay of eight days in 2013. Routine surveillance cultures are taken three times weekly including throat swabs, urine samples and lower respiratory tract specimens for ventilated patients.

MRSA screening from nose, throat, skin and wounds is performed for every patient admitted from another hospital. MRSA positive patients are isolated and contacts are screened. The first MRSA isolate of every positive patient is archived. Patients with previous MRSA history are isolated for the whole hospital stay, even with a negative admission or follow-up screening. Cohort isolation is considered if multiple patients are MRSA positive.

### Epidemiological investigation

#### Definitions

Cases were defined as patients with at least one hospital stay at the relevant department between 01.01.2012 and 18.02.2014 and a positive documentation of *S*. *aureus* resistant to oxacillin/methicillin. Cases were classified as historical cases (HC: patients with previous MRSA history), imported cases (Imp-C: first MRSA result within 48 hours of hospitalization) and hospital-acquired cases (HAC: first MRSA result after 48 hours of hospitalization). If a classification was not possible, cases were categorized as unknown (UNK).

The expression ‘study period’ refers to the time from 01.01.2012 to 18.02.2014; ‘outbreak period’ refers to the case accumulation in 2014 only.

#### Description of cases, hospital stay characteristics and MRSA isolates

Case finding and data acquisition were achieved by active screening of the hospital database and patient records. Obtained variables included characteristics of patients (age, sex), hospital stay (number, time and ward of hospitalization) and MRSA isolates (specimen, time and place of the first isolate). Based on isolate and hospital stay characteristics, cases were further defined by case category (HAC / Imp-C / HC / UNK) and identification procedures (MRSA screening/surveillance culture/clinical investigation). An epidemiological curve with weekly intervals was generated for the analysis of MRSA transmission, and hospital stay characteristics of each case were summarized by time and place of hospitalization.

#### Informed consent and data protection

Data related to human subjects were analysed as part of the routine infection control and outbreak management practices. No samples were obtained in addition to those derived from routine procedures, which were for the patients’ benefits. All data and results are reported anonymously; therefore no specific informed consent was required.

### Microbiological investigation

#### Antimicrobial susceptibility

Susceptibility to oxacillin (OXA), chloramphenicol (CMP), tetracyclin (TET), cefoxitin (CXT), co-trimoxazole (COT), erythromycin (ERY), gentamycin (GEN), clindamycin (CLI), ciprofloxacin (CIP), vancomycin (VAN), teicoplanin (TEI) and rifampicin (RIF) was determined for MRSA isolates using the disc diffusion test methodology as defined by the European Committee on Antimicrobial Susceptibility Testing (EUCAST) (http://www.eucast.org/zone_diameter_distributions/). Susceptibility patterns in the results are demonstrated in the above mentioned order and illustrated by “R” for resistant and “C” for susceptible.

#### Toxin profile

The expression of the staphylococcal toxic shock syndrome toxin (TSST-1), staphylococcal enterotoxins A-E (SET-A, -B, -C, -D, -E) and exfoliative toxins A and B (ETA/ETB) was determined by commercial reversed passive latex agglutination tests according to the manufacturers’ instructions (TST-RPLA Kit, Oxoid; SET-RPLA Kit, Oxoid; EXT-RPLA “Seiken”, Denka-Seiken Co., LTD). For the detection of the Panton-Valentine leukocidin (PVL) and *mecA* genes, PCR assays were performed as described previously [12,13]. The production of α-, β- and δ-haemolysin was characterised on blood agar based on either synergy or antagonism with β-haemolysin of the *S*. *pseudintermedius* standard strain CCM 4710 [14,15].

#### Phage typing

For the assignment of individual phage types, the established standard method [10] and an international set of 26 phages from Public Health England (Colindale, UK) and the Robert Koch-Institute (Wernigerode, Germany) were applied.

#### Spa typing

The amplification of the *Staphylococcus* protein A gene (spa) was done using the primer pair spa-1113f and spa-1514r [16]. All other steps related to laboratory procedures and sequence analyses were performed as described elsewhere (http://www.seqnet.org/pdf/Sequencing_procedure_lab.pdf) [17].

#### Pulsed-field gel electrophoresis (PFGE) typing

PFGE profiles based on *Sma*I restriction patterns were conducted as described previously [18].

### Investigation of work-related factors

#### Audit of nursing, infection control and general work practices

Audits were performed by two specialised infection control nurses who worked four shifts each at the ICU, independently of each other and at three different time periods (day, night and weekend). During these shifts, factors related to quality of nursing, management, general work procedures and infection control practices were observed and rated in a five-membered scale. The level of compliance was described as 1 = 0–20%, 2 = 21–40%, 3 = 41–60%, 4 = 61–80% and 5 = 81–100% (S1 File).

#### Analysis of work conditions and psychological aspects

Work-related, psychological and behavioural factors with a possible impact on the quality of work were analysed by interviewing ICU nurses in anonymous questionnaires (S2 File). Questions addressed the personal sensation towards compliance with hygiene and work procedures by ICU staff, interpersonal relations, stress level, subjective impression on number of staff and motivation to work. The analysis also investigated the trend towards improvement or degradation of these factors within three month prior to the outbreak.

## Results

### Epidemiological investigation

Thirty one cases matching the case definition were identified (Table 1). The mean age was 71y (range 50y–88y) and 52% of cases were male. MRSA strains were initially isolated from sputum or endotracheal aspirate (23%), wound swabs (19%), skin (19%), nose (23%), blood (6%) and other specimens (10%). Identification of MRSA occurred in 45% of cases by targeted screening procedures and 58% were identified at the ICU for the first time. 52% (16/31) of cases were classified as HAC, 26% (8/31) as Imp-C, 13% (4/31) as HC and 10% (3/31) as UNK cases (Table 1).

**Table 1:** Epidemiological description of MRSA cases, 01/2012–02/2014 (ICU = intensive care unit; SW = standard ward; ETA = endotracheal aspirate; HC = historical case; Imp-C = imported case; HAC = hospital acquired case; UNK = unknown; CV = central venous catheter).

Case	Date of first detection	Place of first detection	Specimen	Identification procedure	Classification
1	20120201	ICU	Skin	MRSA Screening	HC
2	20120301	SW	Sputum/ETA	Clinical	Imp-C
3	20120314	SW	Wound	Clinical	HAC
4	20120319	ICU	Skin	MRSA Screening	HAC
5	20120322	SW	Wound	Clinical	Imp-C
6	20120406	ICU	Urogenital	Surveillance culture/clinical	HAC
7	20120508	SW	Sputum/ETA	Clinical	HAC
8	20120522	SW	Skin	MRSA Screening	HC
9	20120528	ICU	Sputum/ETA	Surveillance culture/clinical	HAC
10	20120528	ICU	Skin	MRSA Screening	Imp-C
11	20120525	OTHER	Blood	Clinical	Imp-C
12	20110114	SW	Sputum/ETA	Clinical	Imp-C
13	20120625	ICU	Nose	MRSA Screening	Imp-C
14	20121208	SW	Sputum/ETA	Clinical	Imp-C
15	20130415	ICU	Sputum/ETA	Surveillance culture/clinical	HC
16	20130610	SW	Skin	MRSA Screening	HC
17	20130723	ICU	Wound	Clinical	UNK
18	20130724	ICU	Wound	Clinical	HAC
19	20130725	OTHER	Nose	MRSA Screening	UNK
20	20130926	ICU	Wound	Clinical	HAC
21	20131003	ICU	Skin	MRSA Screening	HAC
22	20131106	ICU	Catheter (CV)	Surveillance culture/clinical	HAC
23	20131125	SW	Throat	MRSA Screening	Imp-C
24	20131218	OTHER	Nose	MRSA Screening	UNK
25	20140120	ICU	Sputum/ETA	Surveillance culture/clinical	HAC
26	20140130	ICU	Wound	Clinical	HAC
27	20140203	ICU	Nose	MRSA Screening	HAC
28	20140203	ICU	Nose	MRSA Screening	HAC
29	20140203	ICU	Nose	MRSA Screening	HAC
30	20140203	ICU	Blood	Clinical	HAC
31	20140208	SW	Nose	MRSA Screening	HAC

**Fig 1:**
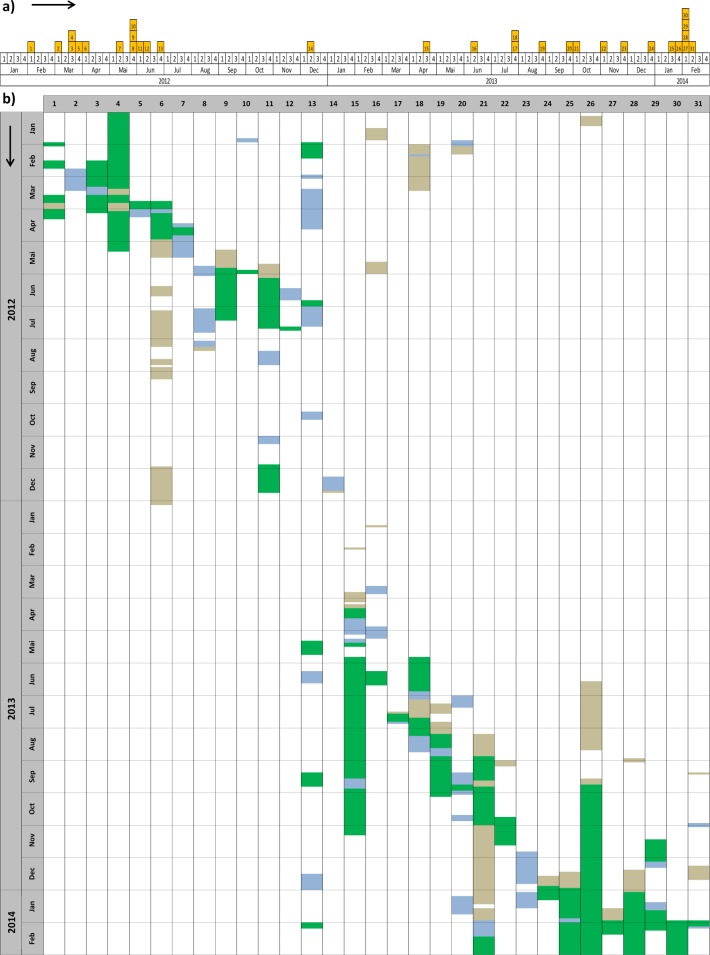
Epidemiological curve (a) and hospitalisation history (b) of MRSA positive patients in the affected department, 2012–2014. Place of hospitalisation is indicated: green = ICU; blue = SW; beige = other department.

The time distribution of MRSA cases revealed a first accumulation of cases between February and June 2012 (Fig 1A). Between July 2012 and June 2013 cases were registered only sporadically. Since July 2013, the number of cases accumulated again, resulting in a peak early 2014. The analysis of hospital stay characteristics revealed overlapping hospitalisations of cases for long periods of time, especially in the ICU ward (Fig 1B).

### Microbiological investigation

Thirty six MRSA isolates obtained from patients, staff and environment were characterised (Table 2). No subtyping was performed for cases 3 and 23 due to missing isolates. Due to unrelated spa types, PFGE types were not determined for cases 8, 10, 11, 14 and 17.

**Table 2:** Microbiological investigation of MRSA isolates, 01/2012–02/2014 (PVL = Panton-Valentine Leukocidin, spa = *S*. *aureus* protein A, PFGE = pulsed-field gel electrophoresis, n.a. = not applicable, R = resistant, C = susceptible, NT = non-typable, hyper = hyperproduction).

	Antibiotic profile	Toxin profile			Subtype		
Case/Isolate	Susceptibility pattern	Haemolysin	Entertoxin	PVL	spa type	PFGE type	phage type
1	RCCRCRCRRCCC	α	D	–	t014	A1	47,54,77,81,812,D11
2	RCCRCRCRRCCC	α	–	–	t003	A1	812
3	RCCRRRCRRCCC	n.a.	n.a.	n.a	n.a.	n.a.	n.a.
4	RCCRCRCRRCCC	α	D	–	t014	A1	6,47,54,75,812,D11
5	RCCRCRCRRCCC	α	D	–	t014	A5	6,42E,47,53,54,75,77,83A,81,812,D11
6	RCCRRRCRRCCC	α	D	–	t014	A1	6,47,54,75,812,D11
7	RCCRRRCRRCCC	α	D	–	t014	A1	6,47,53,54,75,812,D11
8	RCCRCRCRRCCC	α	D	–	t586	n.a.	6,47,54,812,D11
9	RCCRRRCRRCCC	α	D	–	t014	A1	6,47,54,75,812
10	RCCRCCCCCCCC	α	–	–	t164	n.a.	3A,71,812
11	RCCRCRCRRCCC	α	–	+	t008	n.a.	52,79,80,53,83A,85,95,812
12	RCCRCRCRRCCC	α	D	–	t003	A1	6,47,53,54,75,812
13	RCCRCRCRRCCC	α	D	–	t014	A3	75,812
14	RRRRCRRRRCCC	α	A hyper	–	t008	n.a.	NT
15	RCCRCRCRRCCC	α	D	–	t003	A2	6,47,53,54,75,83A,812,D11
16	RCCRCRRRRCCC	α	D	–	t003	A1	75,812
17	RCCRCRCRRCCC	-	D hyper	–	t002	n.a.	812
18	RCCRRRCRRCCC	α	D	–	t014	A1	6,42E,47,53,54,75,77,81,812,D11
19	RCCRCRCRRCCC	α	D	–	t003	A5	6,47,53,54,75,77,83A,812,D11
20	RCCRCRCRRCCC	α	–	–	t003	A1	6,47,54,75,812
21	RCCR(C/R)RCRRCCC	α	D	–	t1282	A1	6,42E,47,53,54,75,77,81,812,D11
22	RCCRCRCRRCCC	α	D	–	t1282	A1	6,47,54,75,812
23	RCCRCRCRRCCC	n.a.	n.a.	n.a.	n.a.	n.a.	n.a.
24	RCCRCRCRRCCC	α	D	–	t003	A4	54,812
25	RCCRCRCRRCCC	α	D	–	t003	A4	54,812
26	RCCRCRCRRCCC	α	D	–	t003	A4	54,812
27	RCCRCRCRRCCC	α	D	–	t003	A4	54,812
28	RCCRCRCRRCCC	α	D	–	t003	A4	54,812
29	RCCRCRCRRCCC	α	D	–	t003	A4	54,812
30	RCCRCRCRRCCC	α	D	–	t003	A4	54,812
31	RCCRCRCRRCCC	α	D	–	t003	A4	54,812
Staff-A	RCCRCRCRRCCC	α	D	–	t014	A1	6,47,53,54,75,77,812,D11
Staff-B	RCCRCRCRRCCC	α	D	–	t014	A1	6,47,53,54,75,77,83A,81,812,D11
Staff-C	RCCRCRCRRCCC	α	D	–	t003	A4	54,812
Tissue box	RCCRCRCRRCCC	α	D	–	t003	A4	54,812
Wheelchair	RCCRCRCRRCCC	α	D	–	t003	A4	54, (77vw), 812
Shampoo	RCCRCRCRRCCC	α	D	–	t003	A4	54, (77vw), 812
Ultrasound	RCCRCRCRRCCC	α	D	–	t003	A4	54,812

All MRSA isolates carried the *mecA* gene and were negative for the production of TSST-1 and exfoliative toxins. All but one isolate were positive for the production of α-haemolysin. 86% (31/36) expressed enterotoxin D and one isolate carried the *pvl* gene (Table 2).

Antimicrobial susceptibility testing revealed one predominant pattern among the tested isolates (RCCRCRCRRCCC) (Table 2). In 2012 and 2013, nine isolates presented different patterns, whereas since 10/2013 all isolates, including staff and environmental isolates, presented the dominant pattern.

**Fig 2:**
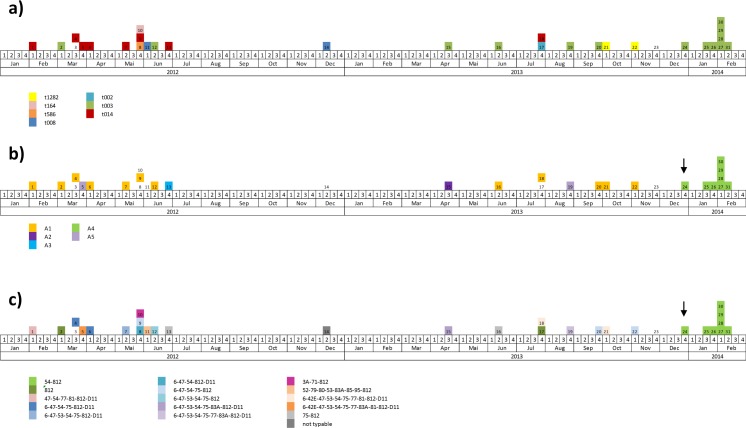
Epidemiological curve according to spa type (a), PFGE type (b) and phage type (c). Numbers in boxes refer to individual cases as listed in Table 2. Arrows indicate the probable index case.

Seven different spa types were identified, of which two appeared predominantly. spa type t003 mainly circulated in 2013 and 2014 and all outbreak-related isolates were of this type. spa type t014 was the predominant strain in 2012 (Table 2 and Fig 2A).

Five different PFGE subtypes were identified. Subtype A1 was the predominant type in 2012 and 2013. PFGE subtype A4 first appeared with case 24 and was allocated to all outbreak-related cases and environmental isolates, as well as one staff isolate (Table 2 and Fig 2B). Subtypes described as A2, A3 and A5 occurred only sporadically.

Phage typing revealed a high diversity of subtypes in the years 2012 and 2013, but only one specific type circulating in 2014 (phage type 54/812) (Table 2 and Fig 2C). Type 54/812 first appeared in the department in December 2013 with case 24. All outbreak-related patient isolates, environmental samples and one staff isolate exhibited the same phage type (Table 2).

Taken together, the bacterial strain associated with the outbreak in 2014 was defined as *S*. *aureus* spa type t003, PFGE type A4 and phage type 54/812. This strain exhibited resistance to oxacillin/methicillin, cefoxitin, erythromycin, clindamycin and ciprofloxacin, and expression of α-haemolysin and enterotoxin D.

### Investigation of psychological, behavioral and work-related factors

**Fig 3:**
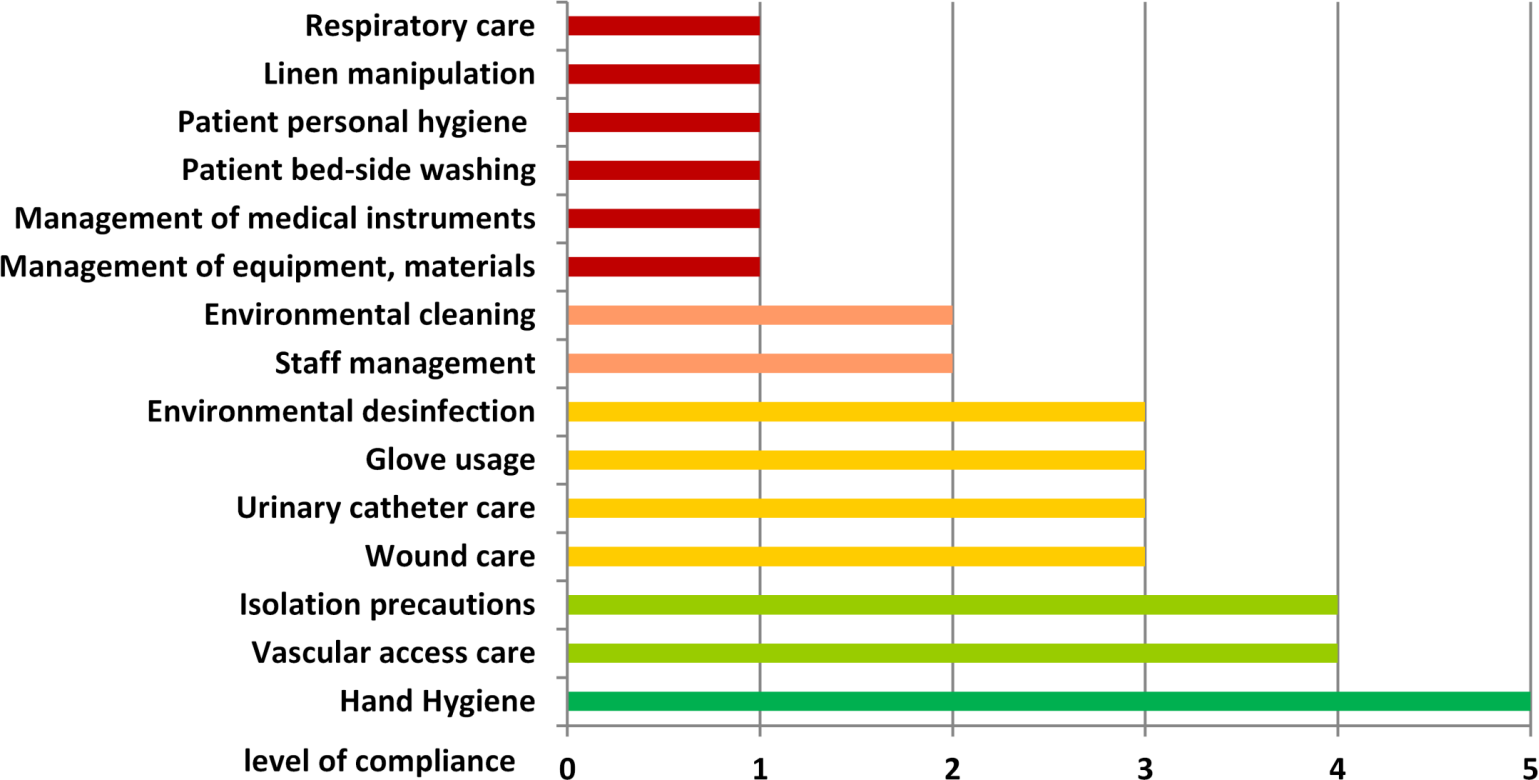
Analysis of work related factors, management and nursing procedures by impartial audits performed by specialized infection control nurses (audit form: S1 File).

Audits performed at the ICU revealed important gaps in infection control practices (Fig 3). Most critical parameters were observed in general management and nursing procedures, with focus on personal patient hygiene. Compliance with hand hygiene and isolation precautions for MRSA positive patients were rated as appropriate. The results of both infection control nurses were in agreement.

**Fig 4:**
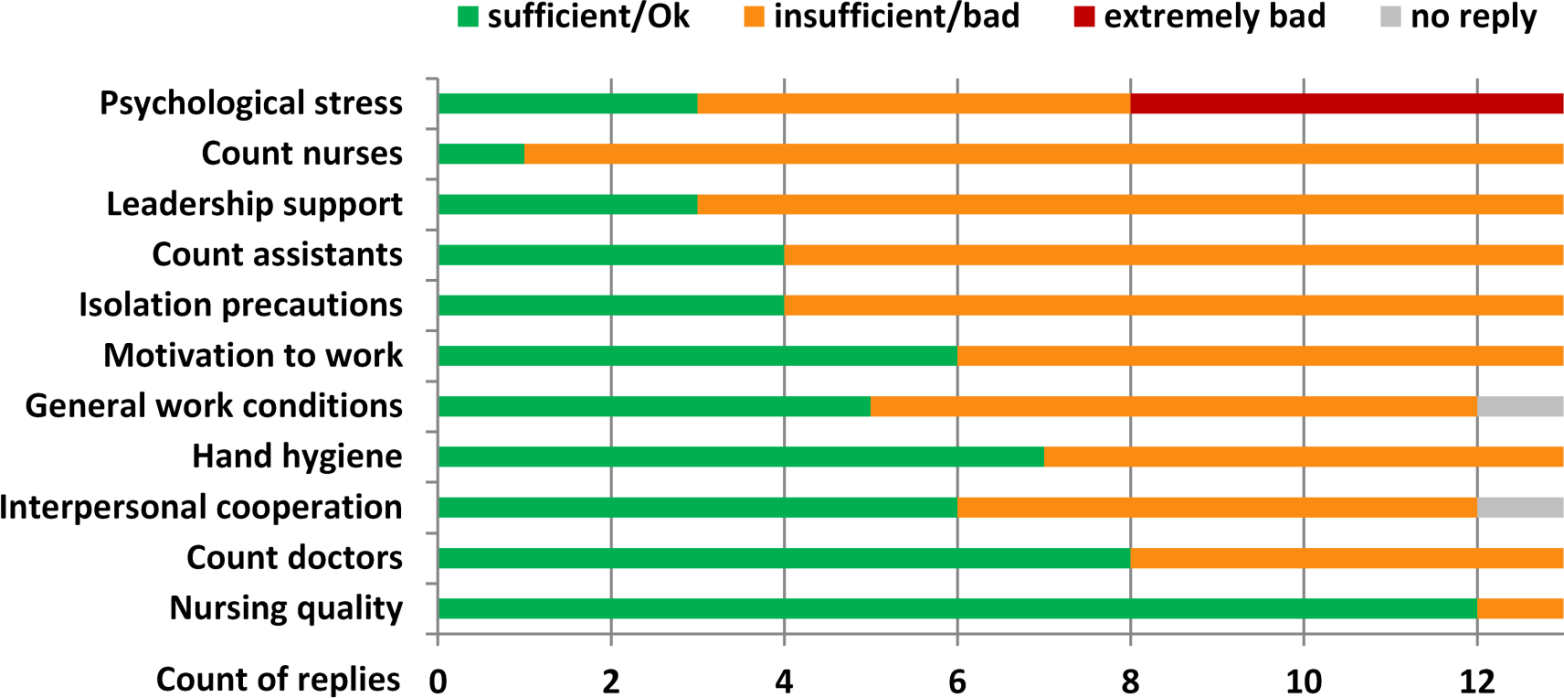
Investigation of work-related and psychological factors by nurse interviews (questionnaire: S2 File).

General work conditions and psychological factors with potential impact on the work performance were investigated by interviewing ICU nurses. The response rate was 72% (13/18). Nearly all factors addressed were rated as ‘insufficient’, ‘bad’ or ‘extremely bad’ by at least 50% of the participants (Fig 4). The number of nurses was rated as ‘insufficient’ by 92% (12/13), followed by the support of the leadership (rated as ‘insufficient’ by 77% (10/13)) and the intensity of psychological stress (rated as ‘high’ or ‘extremely high’ by 77% (10/13)). An exception was the quality of nursing, which was valued as ‘sufficient’ by 12 out of 13 nurses (92%). More than 50% of the nurses experienced worsened or extremely worsened conditions related to stress level, general working conditions and support from the leadership within a three-months’ time period prior to the outbreak in 2014 (data not shown).

## Discussion

The outbreak strain and the probable index case for the described outbreak were identified by epidemiological and microbiological analyses. The index patient was admitted from a long term care facility with continuous MRSA problems, implying importation of the strain even though a case classification was not possible. All subsequent cases in 2014 were classified as HAC, had overlapping hospitalisation at the ICU and carried the same bacterial strain, verifying the outbreak incidence. The evidence of the same strain in environmental samples and a staff’s nasal swab identified the most probable bacterial reservoirs and transmission routes. The proportion of 29% environmental MRSA contamination (4/14 samples) was very high and was mainly present on items used by HCW for patient hygiene. De Lassance reported an outbreak with ongoing *S*. *aureus* transmission due to an environmental contamination of up to 14%, which mainly occurred outside the patient rooms [19]. In both situations the role of staff in pathogen transmission is evident. In the present study, the staff colonised with the outbreak strain was a new employee and presumably got colonised through inappropriate nursing of the index case. However, nosocomial outbreaks promoted by MRSA colonised HCWs are mostly associated with incorrect nursing practices rather than asymptomatic nasal carriage itself [20–22]. This is in agreement with the presence of two other employees at the department, who were knowingly MRSA positive for a long period of time, but not related with one of the cases within the last two years as indicated by microbiological typing. Frequent and extensive staff education should therefore be a key preventive measure to limit the risk of MRSA cross-transmission by HCWs.

The analysis of procedures involved in MRSA case identification revealed that the applied risk based screening design was not sufficient to detect all cases and prevent further transmission. Only 52% of cases were identified by targeted screening procedures. Furthermore, screening procedures were not fully compliant with the hospital guidance, which poses challenges for case identification, categorisation and patient management. Underestimation and silent transmission of MRSA can therefore especially be assumed on SW, where no routine screening is in place. Replacing the screening strategy of high-risk patients by a screening approach of high-risk units might be considered to improve infection control at the affected ICU, as recommended for healthcare settings with significant problems [23].

A set of different microbiological and molecular methods was used for the characterisation of MRSA isolates. Antimicrobial susceptibility and toxin profiles were rather homogenous, which is expected for bacterial populations from single hospital settings [24]. spa typing revealed two predominant types among the investigated isolates (t003 and t014), but could not clearly indicate the source of the 2014 outbreak. Furthermore, both types are genetically related and emergence of one type from the other over time cannot be excluded. Due to its insufficient discriminatory power for nosocomial outbreak situations, spa typing is generally recommended for superregional surveillance purposes [11]. PFGE typing also showed mainly two subtypes circulating within the study period (A1 and A4). In this case however, the outbreak related cases in 2014 were clearly separated from the cases in 2012 and 2013. This method presented a great value regarding source identification, but could not resolve individual strains in the years 2012 and 2013. In non-outbreak situations, this might lead to misinterpretation in the presence of consistent bacterial populations. Considering PFGE results alone, the cases in 2012 and 2013 would be interpreted as related and the staff colonised with the same PFGE subtype could wrongly be determined as the source for ongoing transmission. Similar to PFGE typing, phage typing was suitable for source identification of the 2014 MRSA outbreak. Additionally, it revealed a high diversity of different subtypes in the time period before the outbreak. Even though rarely performed in these times, phage typing showed the highest discriminatory power in this study and proved to be a useful alternative to modern typing methods for the investigation of nosocomial MRSA outbreaks.

The hospitalisation history of cases argued for two independent MRSA outbreaks in 2012 and 2013/2014, which was not supported by other epidemiological and microbiological results. The diversity of MRSA strains in 2012/2013 identified by phage typing argues against continuous transmission. However, taking genetic evolution and the exchange of virulence factors into account, which has been described to occur even within individual outbreaks, small differences in typing results would not necessarily exclude a link between cases, especially when observed over long time periods [24,25]. There are no fixed guidelines available which regulate the assignment of new bacterial subtypes. The designation has to be made rather on an individual basis, depending on factors such as the discriminatory power of the method, the epidemiological context or the geographical and temporal distribution of isolates. The combined application of different typing methods might therefore be preferential, as supported by the conflicting typing results in this study [8,9,11].

The investigation of psychological and work-related factors revealed important gaps in infection control and management practices. The results addressing the quality of patient care were partially controversial between staff self-evaluations and impartial audits, and demonstrated the need for reinforced staff education.

The most worrisome outcomes of nurse interviews were the tremendous psychological pressure, a low motivation to work and the presence of interpersonal conflicts, reflected by absence of leadership support and cooperation. Appropriate work conditions are of utmost importance in psychological stressful positions such as ICU work. Poor conditions, stress and insufficient communication have a direct impact on the quality of nursing and may have severe consequences for the patients [26–31]. Especially the care for patient populations with chronic diseases and poor prognoses, as described here, can easily lead to exhaustion and frustration if not addressed properly.

Even though the number of nurses was rated as insufficient by nurse evaluations, the determined nurse-to-patient ratio was 0.4–0.5 during all shifts, which is in line with the recommended conditions for ICU settings [32]. The audits revealed important gaps in general management procedures, including missing work schedules, undefined personal responsibilities, insufficient supervision as well as the inappropriate management and use of materials and medical equipment. Major misbehaviour was also observed in nursing procedures such as incorrect performance of bed-side toilet for immobile patients, incomplete linen exchange after body fluid contamination and incorrect care of wounds and invasive devices. In contrast, compliance with hand hygiene and contact precautions for MRSA positive patients was appropriate and according to standard recommendations [33]. However, hand hygiene compliance was not measured in relation to the number of hand hygiene opportunities and thus cannot be evaluated in a quantifiable way. Furthermore, a limitation of this analysis is the lack of information on hand hygiene compliance before and during the outbreak period. Audits were performed openly after discussion of the outbreak situation, and therefore a Hawthorne effect cannot be excluded.

Investigations of nosocomial MRSA outbreaks usually include epidemiological and microbiological methods or focus on well-described problem areas such as hand hygiene compliance [19–21,34–39]. Managerial or psychological aspects are less frequently taken into account. However, this study demonstrates that these neglected factors play a crucial role in infection control, and more studies investigating the direct impact of work-related and psychological factors on the development of nosocomial outbreaks are needed. Behavioural and psychological studies, as well as the evaluation of managerial components should therefore find their way in routine infection control strategies and outbreak investigations. A set of possible interventions has been reviewed recently [40].

## Conclusion

This investigation highlights the importance of examining nosocomial outbreaks in a multi-faceted approach, comprising epidemiological, microbiological, psychological and behavioural disciplines.

Different factors promoted the described outbreak, including the introduction of a new employee at times of increasing stress levels and worsening working conditions, reflected by major gaps in managerial processes and communication. In return, the existence of a microbiological surveillance and infection control capacity facilitated the outbreak detection, the early response and control. However, this alone doesn’t prevent the emergence of nosocomial outbreaks. Implementing preventive routine activities addressing work-related, psychological and behavioural factors is crucial to improve infection control in long term, as shown in this study. Regular evaluations of work conditions and performance, continuous staff education as well as provision of training in stress, conflict and general management should be considered in order to increase patient safety permanently, especially in patient populations with poor prognosis.

## Supporting Information

S1 File Audit form. Analysis of nursing procedures, operational management and infection control measures
(PDF)

S2 File Questionnaire. Analysis of psychological and work-related factors
(PDF)
