# Supplementary material for: Importance of Multifaceted Approaches in Infection Control: A Practical Experience from an Outbreak Investigation
Source: PLoS One. 2016 Jun 20;11(6):e0157981. doi: 10.1371/journal.pone.0157981 (PMC4913898; doi:10.1371/journal.pone.0157981)
Supplement: S1 File — Analysis of nursing procedures, operational management and infection control measures (PDF) [file pone.0157981.s001.pdf]

S1 File. Audit form. Analysis of nursing procedures, operational management and infection control measures

Observed item	Compliance					Comments
	1 (≤20%)	2 (21-40%)	3 (41-60%)	4 (61-80%)	5 (81-100%)	
**Hand hygiene and use of gloves**						
hand disinfection						
use of gloves						
**Isolation of MRSA positive patients**						
contact precautions						
**Nursing procedures**						
personal hygiene of patients						
bed-side toilet of patients						
linen manipulation						
respiratory care						
vascular access care						
wound care						
urinary catheter care						
**Operational management**						
nurses and aides management						
use of medical instruments						
use of equipment, materials						
use of medicines						
**Environmental cleaning**						
environmental cleaning						
environmental disinfection						
