# Supplementary material for: Importance of Multifaceted Approaches in Infection Control: A Practical Experience from an Outbreak Investigation
Source: PLoS One. 2016 Jun 20;11(6):e0157981. doi: 10.1371/journal.pone.0157981 (PMC4913898; doi:10.1371/journal.pone.0157981)
Supplement: S2 File — Analysis of psychological and work-related factors (PDF) [file pone.0157981.s002.pdf]

S2 File. Questionnaire. Analysis of psychological and work-related factors

1. A) How do you rate the compliance with hand hygiene of healthcare workers (HCW) in the ICU?
   Sufficient ☐ Insufficient ☐ Irrelevant ☐

   B) Did you observe a trend over the last three months?

   Unchanged ☐ Improved ☐ Worsened ☐ Extremely worsened ☐

2. A) How do you rate the compliance with isolation precautions of HCW with MRSA positive patients?
   Sufficient ☐ Insufficient ☐ Irrelevant ☐

   B) Did you observe a trend over the last three months?

   Unchanged ☐ Improved ☐ Worsened ☐ Extremely worsened ☐

3. A) How do you rate the quality of nursing in the ICU?
   Sufficient ☐ Insufficient ☐ Irrelevant ☐

   B) Did you observe a trend over the last three months?

   Unchanged ☐ Improved ☐ Worsened ☐ Extremely worsened ☐

4. A) How do you rate your experience on the cooperation between doctors and nurses in the ICU?
   Good ☐ Bad ☐ No reply ☐

   B) Did you observe a trend over the last three months?

   Unchanged ☐ Improved ☐ Worsened ☐ Extremely worsened ☐

5. A) How do you rate the working conditions in the ICU in general?
   Good ☐ Bad ☐ No reply ☐

   B) Did you observe a trend over the last three months?

   Unchanged ☐ Improved ☐ Worsened ☐ Extremely worsened ☐

6. A) Do you feel support from the leadership related to your work?
   Sufficient ☐ Insufficient ☐

   B) Did you observe a trend over the last three months?

   Unchanged ☐ Improved ☐ Worsened ☐ Extremely worsened ☐

7. How do you rate the number of nurses at the ICU?
   Sufficient ☐ Insufficient ☐

8. How do you rate the number of doctors at the ICU?
   Sufficient ☐ Insufficient ☐

9. How do you rate the number of assistants at the ICU?

Sufficient ☐ Insufficient ☐

10. A) How do you rate your experience on the amount of psychological stress among HCW at the ICU?
    Adequate ☐ High ☐ Extremely high ☐

    B) Did you observe a trend over the last three months?
    Unchanged ☐ Decreased ☐ Increased ☐ Extremely increased ☐

11. A) How is your motivation to work?
    Good ☐ Bad ☐ Burn out conditions ☐

    B) Did you observe a trend over the last three months?
    Unchanged ☐ Improved ☐ Worsened ☐ Extremely worsened ☐
